# The Diagnostic Pitfalls and Clinical Challenges of Unilateral Facial Paralysis in Acute Demyelinating Disorders: A Case Report and Literature Review

**DOI:** 10.1155/crnm/7635056

**Published:** 2025-10-07

**Authors:** Thamer S. Alhowaish, Hossam Ali Alqahtani, Moustafa S. Alhamadh, Ali Alanazi

**Affiliations:** ^1^Department of Neurology, Ministry of National Guard-Health Affairs, Riyadh, Saudi Arabia; ^2^King Abdullah International Medical Research Center, Riyadh, Saudi Arabia; ^3^King Saud bin Abdulaziz University for Health Sciences, Riyadh, Saudi Arabia; ^4^Department of Medicine, Ministry of National Guard-Health Affairs, Riyadh, Saudi Arabia

**Keywords:** Bell's palsy, demyelination, Guillain–Barré syndrome, peripheral neuropathy, unilateral facial palsy

## Abstract

Guillain–Barré syndrome (GBS) is an acute immune-mediated polyradiculoneuropathy typically presenting with progressive limb weakness and areflexia, while bilateral facial nerve involvement is a well-recognized feature. However, unilateral facial palsy is exceedingly rare and can closely mimic Bell's palsy, complicating early diagnosis. We report the case of a previously healthy 32-year-old man whose illness began with subtle bilateral fingertip numbness ascending to his elbows, followed by the acute onset of right-sided facial weakness, perioral numbness, slurred speech, and inability to close his right eye. These symptoms developed shortly after an upper respiratory tract infection and were soon accompanied by toe numbness and gait unsteadiness. Examination revealed isolated right lower motor neuron facial palsy and a rapid progression from diminished to absent deep tendon reflexes, while muscle strength and general sensation remained preserved. The diagnosis of GBS was confirmed by absent reflexes, albuminocytologic dissociation in cerebrospinal fluid, and electrodiagnostic evidence of bilateral facial and trigeminal neuropathy. The patient was treated successfully with intravenous immunoglobulin, resulting in significant clinical improvement. This case underscores the diagnostic challenges of atypical GBS presentations and highlights the importance of considering GBS in patients with acute, evolving cranial neuropathies, even when the presentation closely resembles more common conditions such as Bell's palsy.

## 1. Introduction

Guillain–Barré syndrome (GBS) is an acute demyelinating disorder that presents in the classic form of muscle weakness and absent reflexes [1]. A rare variant of GBS includes facial paresthesia and bilateral facial weakness. Because of these atypical manifestations, it is occasionally challenging to diagnose GBS when it presents with this rare variant [2–4]. In general, bilateral facial nerve palsy is rare, with an incidence of only 1 per 5,000,000 population, and only 20% of the cases are idiopathic [5]. A study of 43 patients with bilateral facial nerve palsy found that 10 cases were attributed to Bell's palsy, and five were due to GBS [6]. This variant of GBS typically manifests by numbness in the limbs, followed by bilateral facial nerve palsy. Interestingly, in rare cases, GBS also presents with unilateral facial weakness that is difficult to distinguish from Bell's palsy [2–4, 7]. The pathology of unilateral facial palsy in GBS involves immune-mediated nerve damage, leading to demyelination or axonal injury of the facial nerve. While facial involvement in GBS is typically bilateral, an asymmetric immune response can result in unilateral facial palsy due to localized inflammation [8, 9].

Here, we report a case of a rare variant of GBS with unilateral facial weakness and paresthesia.

## 2. Case Report

### 2.1. History of Presenting Illness

**Figure 1:**
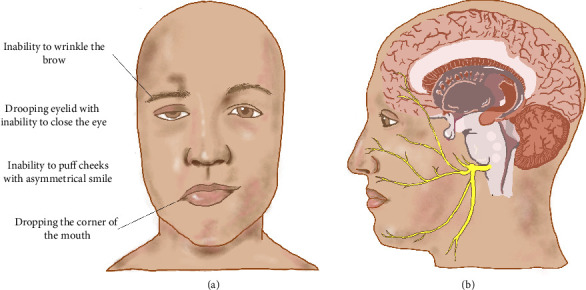
(a) Clinical photograph showing right lower motor neuron facial palsy, consistent with the presentation observed in our patient. (b) Schematic overview of the facial nerve anatomy, illustrating its course from brainstem exit to its terminal branches on the face. Reproduced with permission from Yazan Alghammas as this figure has been drawn specifically for this research project.

A 32-year-old male, not known to have a previous medical history, presented to the emergency department complaining of right-sided facial weakness with perioral numbness for two days. The symptoms started with numbness in the fingertips bilaterally that progressed to the elbows. One week later, he developed slurred speech, facial deviation on the right side, and an inability to close the right eye (Figure 1). Three days later, he developed numbness in his toes associated with unsteadiness. The symptoms were persistent since their onset and had never occurred before. The patient had upper respiratory tract infection (URTI) symptoms 10 days prior to the onset of his neurological symptoms. He denied any earache, hearing difficulty, tinnitus, recent trauma to the head or neck, or weakness in other parts of the body. There was no history of skin rash, tick bite in the past, promiscuous sexual behavior, or use of any herbal supplements.

### 2.2. Clinical Examination

The patient remained hemodynamically stable with normal vital signs. Neurological assessment revealed intact cranial nerves except for right lower motor neuron-type facial weakness (e.g., ipsilateral forehead involvement). Motor examination demonstrated normal tone and strength in all limbs without muscle atrophy. Deep tendon reflexes were globally diminished at admission, progressing to areflexia (brachioradialis, biceps, triceps, patellar, and Achilles tendons) by day 6. Plantar responses were flexor bilaterally. Sensory testing showed intact light touch, pinprick, vibration, and proprioception in all extremities. Cerebellar function including finger-to-nose, heel-to-shin, and rapid alternating movements was normal without dysmetria or intention tremor.

### 2.3. Investigation

Basic laboratory tests were unremarkable except for polycythemia; chest radiographs were normal. Magnetic resonance imaging (MRI) of the brain with gadolinium administration revealed smooth enhancement in the right internal auditory canal and the right geniculate ganglion. Examination of the cerebrospinal fluid (CSF) showed high protein 0.61 g/L (normal range 0.15–0.40 g/L) and a normal range of white blood cells (WBCs) (Table 1). A nerve conduction study (NCS) and blink reflex were performed 2 weeks after the onset of his symptoms which showed a picture of right carpal tunnel syndrome but otherwise unremarkable findings.

To rule out other possible causes of his neurological symptoms, further tests were performed. Cytologic examination of the CSF revealed a few lymphocytes with no malignant cells. Vitamin B12 level was normal. Antibodies to human immunodeficiency virus (HIV), Epstein–Barr virus (EBV), *Cytomegalovirus* (CMV), and EBV early antigen were negative. Blood and CSF cultures were negative. A stool culture was not performed. Analysis of anti-glycolipid antibodies was negative (Table 2).

### 2.4. Management and Outcome

On the 5th day of admission, he was started on intravenous immunoglobulin (IVIG), 0.4 g/kg/day, for five days along with occupational and physical therapy.

The patient was discharged with a diagnosis of atypical GBS. At the 2-week follow-up in the clinic, the facial weakness improved significantly. However, the patient complained of perioral numbness and bilateral upper limbs, up to the elbow, and feet numbness. Also, he had decreased pinprick sensation on the right side over the mandibular nerve distribution, and he remained areflexic all over the body.

**Figure 2:**
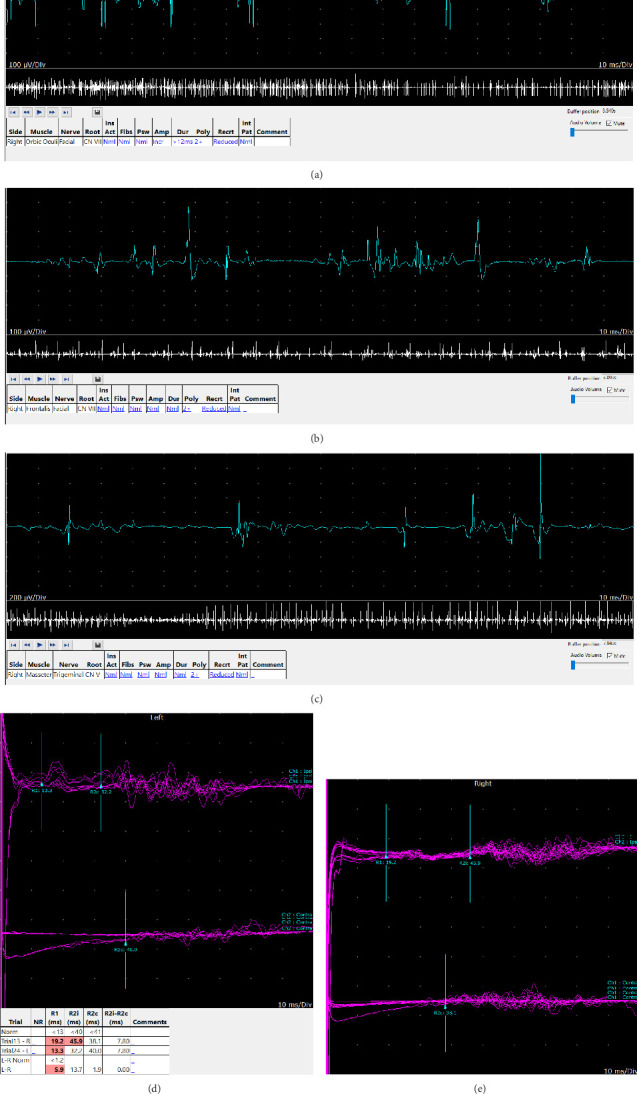
Electromyographic (EMG) and blink reflex studies demonstrating facial and trigeminal nerve involvement. (a) and (b) show EMG responses from the orbicularis oculi and frontalis muscles, respectively, following right facial nerve stimulation. (c) displays EMG recording from the masseter muscle after right trigeminal nerve stimulation. (d) and (e) present blink reflex recordings from the right and left sides. The blink reflexes revealed abnormalities consistent with right facial neuropathy. Needle EMG of bilateral facial and trigeminal nerve–innervated muscles (frontalis, orbicularis oculi, and masseter) revealed mild denervation changes and evidence of chronic neurogenic remodeling.

Three months later, at follow-up, NCS, electromyography (EMG), and blink reflex were performed (Table 3). NCS showed a picture of right median neuropathy, consistent with the previous test. Blink reflexes showed features consistent with right facial neuropathy. Needle EMG of muscles innervated by the facial and trigeminal nerves (frontalis, orbicularis oculi, and masseter) bilaterally demonstrated mild denervation changes as well as chronic neurogenic changes (Table 4 and Figure 2).

The patient underwent multiple follow-up evaluations in the neurology clinic, with the most recent visit occurring 22 months postdischarge. At that time, he had returned fully to his neurological baseline, with complete resolution of facial asymmetry. His only complaint was intermittent tingling sensations in the left arm, which were not associated with any objective neurological deficits. A comprehensive neurological examination was unremarkable, and deep tendon reflexes were symmetrically preserved as 2+ throughout.

## 3. Discussion

In this article, we shared our experience in diagnosing and managing a variant of GBS. Initially, our patient came with unilateral facial weakness and paresthesia following an URTI, because of which he was diagnosed as a case of unilateral Bell's palsy. However, he developed areflexia throughout the body, for which he underwent lumbar puncture and neurophysiological studies (NCS, EMG, and blink reflexes) that showed albumin-cytological dissociation and evidence of facial and trigeminal neuropathy, respectively. The diagnosis of variant GBS with unilateral facial nerve palsy was made based on the clinical examination, CSF analysis, and neurophysiological studies. Treatment of GBS includes supportive measures and disease-modifying therapies such as IVIG or plasma exchange [2]. Our patient responded well to IVIG and had a significant neurological improvement on follow-up visits. While facial weakness can emerge following the resolution of Miller Fisher syndrome (MFS), our patient had no history of diplopia, and the clinical examination showed neither ophthalmoparesis nor ataxia, making the diagnosis of MFS unlikely [9–11].

GBS is classically recognized as a postinfectious polyneuropathy that typically presents with acute lower limb weakness [12]. While this hallmark presentation is well documented, cranial nerve involvement particularly of the facial nerve is also not uncommon feature [7]. When facial involvement occurs, it is characteristically bilateral and often accompanied by distal limb paresthesia or weakness [13]. Interestingly, isolated facial diplegia represents a rare GBS variant, and cases presenting with unilateral facial weakness are exceedingly uncommon, with only a few cases described so far [2, 8] (Table 5). In some reported cases, the weakness started unilaterally and progressed to bilateral facial involvement. However, in our case, it started as and continued to be unilateral while the neurophysiological studies showed evidence of bilateral facial involvement. GBS typically presents with symmetrical neurological deficits; some may question why, in rare cases, facial palsy appears unilaterally such as on the right side rather than the left. While the precise reason remains unclear, several anatomical, immunological, and physiological factors may contribute to this paradoxical presentation. The proposed mechanism involves asymmetric integrity of the blood-nerve barrier (BNB); a more intact BNB on the clinically unaffected side may restrict the infiltration of pathogenic antibodies (e.g., anti-ganglioside antibodies such as GM1) and inflammatory cells, thereby preventing overt clinical symptoms. In contrast, a compromised BNB on the affected side may allow immune-mediated demyelination or axonal injury. Neurophysiological studies often reveal bilateral abnormalities, suggesting subclinical involvement even on the asymptomatic side [8, 9]. Additionally, uneven antigen exposure from prior infections might lead to antibodies targeting specific nerve segments unilaterally, influenced by variations in ganglioside distribution or localized nerve injury [26]. Furthermore, the facial nerve's anatomical pathway through the narrow fallopian canal makes it particularly susceptible to inflammation-induced compression (Figure 1). Asymmetric edema within this confined space may precipitate unilateral facial palsy despite underlying bilateral nerve involvement [8, 26]. These factors collectively illustrate how GBS can present with unilateral facial weakness, highlighting the importance of thorough diagnostic evaluation including NCSs and CSF analysis to ensure accurate diagnosis, particularly in atypical presentations.

The cranial nerves' involvement and enhancement on MRI can be observed in GBS. This was noted in our case as well as in Sharma's case report and Zuccoli's study [16, 27]. Facial nerve enhancement on MRI is observed in both GBS and Bell's palsy but differs in pattern and clinical context. In GBS, enhancement is often bilateral and may involve multiple cranial nerves, reflecting diffuse demyelination and BNB disruption [28]. In contrast, Bell's palsy typically shows unilateral enhancement, most commonly in the labyrinthine segment of the facial nerve, due to localized viral neuritis [29]. While MRI findings may overlap, diffuse or bilateral enhancement supports a diagnosis of GBS, whereas isolated, unilateral enhancement favors Bell's palsy. Thus, clinical correlation remains essential for accurate differentiation.

Deep tendon reflexes are generally absent or diminished. Rare cases without hypo/areflexia have been described, mostly in the acute motor axon neuropathy variant of GBS [6, 30, 31]. In our patient, the reflexes of the upper and lower limbs were absent. A history of infection within four weeks before the onset of neurologic symptoms is found in many patients, and most of them have evidence of demyelination in their limbs on NCS [7, 13]. In the early stages of GBS, neurophysiological assessments such as NCSs may yield negative or inconclusive results. This diagnostic limitation arises from different pathophysiological factors. First, during the initial ≤ 7 days of the disease, the autoimmune response predominantly targets proximal nerve roots and nodes of Ranvier, leading to inflammatory edema and reversible conduction block rather than overt demyelination or axonal injury. Consequently, key electrophysiological parameters such as motor and sensory conduction velocities and compound muscle action potential amplitudes often remain within normal limits until more permanent structural damage, like Wallerian degeneration, emerges typically after 1-2 weeks. Second, early lesions in GBS frequently involve proximal nerve segments, such as spinal roots, which are not readily evaluated by conventional distal-focused NCS techniques. As a result, early diagnostic sensitivity is reduced, underscoring the importance of clinical judgment and potential adjunctive testing in the early phase of GBS [32–34]. The guidance outlined in the paper “*Diagnosis and Management of Guillain*–*Barré Syndrome in Ten Steps*” underscores the diagnostic challenges in early GBS. It emphasizes that both CSF analysis and electrophysiological studies may produce normal or inconclusive results in the initial stages of the disease, thereby necessitating serial testing to capture evolving pathological changes. The paper also highlights the need for heightened clinical awareness of atypical presentations, which may not conform to classic GBS features at onset. Importantly, it reinforces current treatment guidelines by prioritizing IVIG or plasma exchange as first-line therapies, while advising against the use of corticosteroids due to their lack of efficacy and potential to adversely affect recovery [35].

The use of corticosteroids in the treatment of GBS has been a controversial subject and remains a fertile ground of ongoing research. Historically, corticosteroids have been used to reduce inflammation, impede disease progression, and potentially expedite recovery of GBS. However, recent evidence suggests that their efficacy might be limited, and in some cases, they might exacerbate the disease instead [36, 37]. To clarify, randomized controlled trials evaluating the efficacy of corticosteroids in the treatment of GBS revealed no significant benefit; instead, these trials showed that the use of oral corticosteroids worsened GBS outcomes [35, 38]. Because of that, contemporary treatment guidelines for GBS generally recommend against the routine use of corticosteroids [10]. In our case, corticosteroid therapy was initially withheld due to significant diagnostic uncertainty. The patient's presentation, characterized by isolated facial nerve involvement with preserved reflexes and no evidence of limb weakness, ascending paralysis, or autonomic dysfunction, was more suggestive of Bell's palsy or another focal cranial neuropathy than Guillain–Barré syndrome. However, atypical features such as perioral and distal limb paresthesias raised concerns for an alternative diagnosis, warranting caution with corticosteroid use. While steroids are used in Bell's palsy, they are not indicated in GBS. Therefore, corticosteroid administration was deferred due to clinical ambiguity and to allow for further diagnostic evaluation. Ultimately, the evolution of symmetrical areflexia by day six confirmed the diagnosis of GBS. Reflex changes, a hallmark of GBS, often emerge after the onset of motor symptoms, reinforcing the importance of clinical vigilance. Following this diagnostic clarification, GBS-specific treatment was initiated using IVIG. IVIG was selected in this case over plasma exchange due to its similar efficacy, simpler administration, better safety profile, and greater accessibility [35, 39, 40].

GBS with unilateral facial nerve involvement can mimic Bell's palsy, leading physicians to mistakenly administer corticosteroids, which may exacerbate GBS symptoms. Our case, in addition to five published reports, exemplifies the previous scenario, in which Bell's palsy was initially presumed. These five cases initially received corticosteroids for suspected Bell's palsy as shown in Table 5. Distinguishing between Bell's palsy and facial nerve palsy as part of GBS can pose a diagnostic challenge. Bell's palsy presents with an acute onset of one-sided facial nerve palsy, reaching its peak severity in less than 3 days and insidiously resolving over weeks [11]. It often follows a viral infection, particularly herpes simplex virus type 1 and varicella-zoster virus [41]. On the other hand, facial nerve involvement in GBS typically manifests bilaterally, either simultaneously with or preceding the onset of ascending paralysis and paresthesia. This manifestation occurs 1-2 weeks after URTI or gastroenteritis, reaching its peak severity in 2–4 weeks and insidiously subsiding over months to years [42]. In the absence of limb weakness, the presence of high protein levels in the CSF and antiganglioside antibodies helps to distinguish facial nerve involvement in GBS from Bell's palsy [43].

Table 5 summarizes the reported cases of GBS with unilateral facial nerve involvement. We found a total of 23 GBS cases with unilateral facial nerve involvement. The mean age was 33.3 ± 22.9 years (the mean age among adult cases was 44.7 and that among pediatric cases was 7 years), with almost equal gender distribution (12 females and 11 males). Most of the affected patients were healthy with no comorbidities. The majority of the cases were triggered or presumed to be triggered by a respiratory infection or gastroenteritis. More than half (56.5%; *n* = 13) of the reported cases had evidence of bulbar palsy in addition to facial nerve involvement. More than 90% of the cases had diminished or absent deep tendon reflexes. Among the cases where CSF examination was performed, more than two-thirds (68.4%, n = 13) demonstrated albuminocytologic dissociation, defined as elevated CSF protein (> 45 mg/dL) with a total cell count of ≤ 10/mm^3^ [44], and two patients (10.5%) had high protein and slightly elevated WBCs (> 10 and < 20/mm). MRI was negative in almost all cases. IVIG was given for almost three-quarters (73.9%; *n* = 17) of the patients, and some patients received adjunctive therapies such as antiviral therapy. Almost all cases reported a good response to treatment with almost complete recovery; the exception is a 66-year-old female, who was admitted for COVID-19 infection and developed GBS with facial nerve involvement. This patient deteriorated and was admitted to the intensive care unit for respiratory support with multiorgan failure, deep vein thrombosis, and superimposed bacterial infection despite early IVIG and antiviral therapy. Corticosteroids were administered to 5 patients, mainly for presumed Bell's palsy. These patients later received IVIG, and all made a full recovery or had mild residual weakness at the follow-up visit. Our case shares several key features with the 23 previously reported cases of GBS presenting with unilateral facial nerve involvement. Similar to the majority of those cases, our patient was a previously healthy young adult who developed symptoms shortly after an URTI, reinforcing the role of postinfectious immune response as a trigger. The clinical course in our patient also included diminished-to-absent deep tendon reflexes and albuminocytologic dissociation on CSF analysis findings consistent with over 90% and two-thirds of the previously reported cases, respectively. IVIG, which was the most commonly used treatment among the reviewed cases, also resulted in a favorable outcome in our patient. However, our case is distinct in its initial presentation with right facial palsy with perioral numbness, rapidly evolving into broader areflexia without motor weakness, features that added diagnostic complexity. Unlike most prior cases, where bulbar involvement was common (56.5%), our patient did not exhibit bulbar dysfunction. These similarities and differences underscore the heterogeneity of GBS presentations and highlight the need for heightened clinical suspicion in atypical cranial neuropathies.

The disease typically reaches its nadir by 2 weeks in most cases and in 4 weeks in nearly all, after which recovery begins first proximally then distally over weeks or months [13]. Despite a good clinical recovery, many patients exhibit residual weakness and loss of motor units, which likely contributes to the persistent fatigue common in GBS. Early diagnosis and a multidisciplinary rehabilitation program are crucial for improving outcomes. Preclinical studies show that early physical exercise, including active exercise and electrical stimulation, promotes axonal regeneration, prevents maladaptive responses, and upregulates genes linked to neuronal plasticity in both the spinal cord and skeletal muscle. For example, a 12-week study by Chiaramonte et al. demonstrated that bicycle exercise training in GBS patients with severe fatigue was well tolerated and led to marked reductions in fatigue, along with improvements in physical fitness, functional outcomes, and quality of life [45, 46].

This case presents a compelling and educational contribution to medical literature, particularly in the field of neurology, for several key reasons. First, it underscores the critical importance of maintaining a high clinical suspicion for GBS, especially when encountering patients with acute onset unilateral facial weakness, an atypical but possible presentation, particularly when preceded by an infectious illness. Often misattributed to more common conditions such as Bell's palsy, such presentations highlight the necessity of clinicians being familiar with the atypical variants of GBS. Early recognition in these cases allows for prompt and appropriate investigation and treatment, which can significantly impact patient outcomes. Our patient's marked improvement with medical therapy not only reinforces the value of accurate diagnosis but also emphasizes the potential to avoid unnecessary tests and delays in care. Unilateral facial palsy as an initial manifestation of GBS remains rare, as most cases traditionally involve bilateral facial weakness, making this case notable. By drawing attention to this uncommon variant, we aim to broaden the clinical perspective and encourage heightened diagnostic vigilance. This report contributes meaningfully to neurology by bridging a crucial gap in the understanding of GBS presentations, refining diagnostic approaches, and reinforcing the importance of evidence-based management in improving patient outcomes.

## Figures and Tables

**Table 1:** Results of cerebrospinal fluid analysis.

Detail	Value	Normal range
Color of CSF	Colorless	
Appearance CSF	Clear	
WBC CSF	< 1.00	0–5 × 10^6/L
RBC CSF	< 1.00	0–10 × 10^6/L
CSF glucose	4.10	22–3.9 mmol/L
CSF protein	0.61	0.15–0.40 g/L
CSF segs	Not seen	
CSF mono	Not seen	
CSF lymphs	Not seen	
CSF culture	No organisms seen	

**Table 2:** Ganglioside profiles.

	Result	Reference range with unit
MAG-(IgM)	< 30	Up to 30, % AK ratio
GM1 (IgG, IgM)	< 30	Up to 30, % AK ratio
GM2 (IgG, IgM)	< 30	Up to 30, % AK ratio
GD1a (IgG, IgM)	< 30	Up to 30, % AK ratio
GD1b (IgG, IgM)	< 30	Up to 30, % AK ratio
GQ1b (IgG, IgM)	< 30	Up to 30, % AK ratio

**Table 3:** Nerve conduction study and blink reflexes at 3-month follow-up.

**NCS+**									
**Motor nerve results**									
**Site**	**Latency**		**Amplitude**		**F-latency**	**Segment**	**Distance**	**Conduction velocity**	
	**(ms)**	**Normal**	**(mV)**	**Normal**	**(ms)**		**(cm)**	**(m/s)**	**Normal**

*Right median: abductor pollicis brevis muscle (APB)*									
Wrist	**11.4**	< 4.2	**1.53**	> 5.0	**40.3**				
Elbow	15.4		1.27			Elbow-wrist	23	58	> 50
*Right ulnar: abductor digiti minimi muscle (ADM)*									
Wrist	*2.6*	< 4.2	*7.2*	> 3.0	*25.4*				
Below elbow	6.2		6.7			Below elbow-wrist	22	*61*	> 53
Above elbow	7.3		6.6			Above elbow-below elbow	8	*73*	> 52
*Right fibular: extensor digitorum brevis (EDB)*									
Ankle	*4.4*	< 6.1	*2.0*	> 2.0	*48.1*				
Below fibular head	11.5		2.0			Below fib head-ankle	35	*49*	> 38
Popliteal fossa	12.0		2.0			Popliteal fossa-bel fib head	9	*180*	> 42
*Right tibial: abductor hallucis muscle (AHB)*									
Ankle	*3.4*	< 6.1	*6.3*	> 4.4	*46.2*				
Knee	10.0		5.5			Knee-ankle	37	*56*	> 39

**Sensory nerve results**									
**Site**	**Latency (peak)**			**Amplitude (peak-peak)**		**Segment**	**Distance**	**CV**	
	**(ms)**		**Normal**	**(μV)**	**Normal**		**(cm)**	**(m/s)**	**Normal**

*Right median*									
Wrist-digit II	**No response**		< 3.6	**No response**	> 10	Wrist-dig II	14	**No response**	> 39
*Right ulnar*									
Wrist-digit V	*2.5*		< 3.7	**5**	> 15	Wrist-dig V	12	*48*	> 38
*Right radial*									
Forearm-wrist	*1.33*		< 3.1	50		Forearm-wrist	9	68	
*Right sural*									
Calf-lateral malleolus	*1.48*		< 4.0	*74*	> 5	Calf-lateral malleolus	10	*68*	> 35
*Right superficial fibular*									
14 cm-ankle	2.5			*22*	> 5	14 cm-ankle	11	*44*	> 32

**Blink reflexes**									
**Trial**	**NR**		**R1 (ms)**	**R2 ipsilateral (ms)**	**R2 contralateral (ms)**	**R2 ipsilateral-R2 contralateral (ms)**			

Normal			< 13	< 40	< 41				
Right			**19.2**	**45.9**	38.1	7.80			
Left			**13.3**	32.2	40.0	7.80			
Left-right normal			< 1.2						
Left-right			**5.9**	13.7	1.9	0.00			

*Note:* At 3-month follow-up, NCS confirmed right median neuropathy, and blink reflex testing indicated right facial neuropathy.

**Table 4:** EMG at 3-month follow-up.

Side	Muscle	Nerve	Increase activity	Fibrillations	Positive sharp waves	Amplitude	Duration	Polyphasic	Recruitment	Interference pattern
Right	1^st^ dorsal interosseous	Ulnar	Nml	Nml	Nml	Nml	Nml	0	Nml	Nml
Right	Deltoid	Axillary	Nml	Nml	Nml	Nml	Nml	0	Nml	Nml
Right	Masseter	Trigeminal	Nml	Nml	Nml	Nml	Nml	**2**	**Reduced**	Nml
Right	Orbicularis oculi	Facial	Nml	Nml	Nml	**Increase**	**> 12 ms**	**2**	**Reduced**	Nml
Right	Frontalis	Facial	Nml	Nml	Nml	Nml	Nml	**2**	**Reduced**	Nml
Right	Anterior tibialis	Deep fibular	Nml	Nml	Nml	**Increase**	Nml	0	Nml	Nml
Right	Vastus medialis	Femoral	Nml	Nml	Nml	Nml	Nml	0	Nml	Nml
Left	Orbicularis oculi	Facial	Nml	Nml	Nml	Nml	Nml	0	Nml	Nml
Left	Frontalis	Facial	Nml	Nml	Nml	Nml	Nml	0	Nml	Nml
Left	Masseter	Trigeminal	Nml	Nml	Nml	Nml	Nml	0	Nml	Nml

*Note:* Nml: normal. Needle EMG of muscles innervated by the facial and trigeminal nerves (frontalis, orbicularis oculi, and masseter) bilaterally demonstrated mild denervation changes as well as chronic neurogenic changes.

**Table 5:** Summary for the reported cases of GBS with unilateral facial nerve palsy.

Author (year)	Study design	Age and gender	Comorbidities	Possible trigger	Other symptoms/sign	Timing of facial weakness	Side of involvement	Other cranial nerves involvement	Autoantibodies status	MRI findings	CSF findings	Neurophysiology study findings	Treatment	Outcomes
Huang et al. [9] (2021)	Case report and literature review including all GBS/MFS with delayed facial palsy	54 Male	NA	Idiopathic	Lower limbs pain, weakness, and areflexia	3 weeks after lower limbs symptoms	Right	NA	Negative	Negative	WBC count 18 × 10^6^/L, protein 868 mg/L	Reduced amplitude of the facial nerve branches, normal conduction velocity. Facial nerve conduction demonstrated reduced amplitudes in the right facial branches with preserved conduction velocity. Blink reflex showed delayed and reduced R1 and R2 responses on the right compared with the left.	IVIG	The facial nerve completely recovered, but mild lower limbs pain and weakness still present.
Ottaviani et al. [14] (2020)	Case report	66 Female	Hypertension	Coronavirus infection	Difficulty walking, fatigue, and areflexia.	Not mentioned	Not mentioned	NA	Negative	Not done	Albuminocytologic dissociation	Absence of F-waves with diffuse prolonged distal motor latencies and reduced distal compound muscle action potential amplitudes with a slight reduction of conduction velocities	IVIG, antiretrovirals (lopinavir and ritonavir) and hydroxychloroquine.	Not mentioned, but she did not benefit from early IVIG
Chaudhary et al. [15] (2020)	Case report	10 m^ Male	Tricuspid atresia with pulmonary stenosis	Pneumonia	Dysphagia and hyporeflexia	Simultaneous	Left	Bulbar palsy (IX and X CN)	Not done	Not done	Not done	Reduced amplitude, decreased conduction velocity in bilateral peroneal motor nerves, and left tibial nerve. Reduced amplitude in bilateral sural nerves along with reduced amplitude.	Supportive care only	Complete recovery by 4 months
Sharma et al. [16] (2019)	Case report and review of the published cases of unilateral facial palsy in GBS in pediatric population	5 Male	NA	Coronavirus infection	Lower limbs pain, irritability, difficulty walking, loss of balance, generalized hypotonia, and areflexia	4 days prior to the weakness of lower limbs	Left	Bulbar palsy	Not done	Enhancement of the left X and XI and anterior and posterior cervical nerve roots with gadolinium	Albuminocytologic dissociation	Not done	Oral prednisolone then IVIG	The facial nerve recovered and his respiratory status, vocalization, and swallowing improved. However, he still required assistance during walking
Zheng et al. [17] (2018)	Case report	58 Male	Recovered schistosomiasis	Acute hepatitis E	Fatigue, anorexia, cough, mild jaundice, and excretion of tea-colored urine followed by muscle weakness on the lower limbs, numbness, and areflexia	Simultaneous	Right	Bulbar palsy	Negative	Negative	Albuminocytologic dissociation	It showed evidence of demyelinating neuropathy with dysfunction of motor and sensory nerve fibers, but no details were provided.	IVIG and methylprednisolone	On 6-month follow-up, muscle power returned to normal, but there was residual weakness in his right arm.
Nishiguchi et al. [2] (2017)	Case report	54 Male	Ex-smoker	URTI	Paresthesia of the extremities and areflexia.	4 days after the paresthesia	Left	NA	Anti-galactocerebroside and phosphatidic acid were positive	Negative	WBC count 16/μL Protein 71.7 mg/dL, CSF analysis repeated 4 days later showed WBC count 9/μL Protein 72.7 mg/dL	Mildly prolonged distal motor latencies and mildly reduced distal motor amplitude with poor responses in both legs. Bilateral sural nerves showed markedly reduced sensory nerve action potentials	Oral prednisolone, valacyclovir, and methylcobalamin then IVIG	The facial nerve almost completely recovered, and the sensation recovered by 5-month follow-up
Kim et al. [18] (2016)	Case series of 11 patients with acute bulbar palsy as a variant of GBS, 6 of whom had unilateral facial palsy	20 Female	NA	Diarrhea	Dizziness, diplopia, ophthalmoplegia, gait ataxia, sensory symptom, and areflexia	Not mentioned	Not mentioned	Bulbar palsy	IgG anti-GQ1b and GT1a	NA	Unremarkable	Abnormal H-reflexes and blink reflexes, but no details were provided	IVIG	Cured by 6 weeks
Kim et al. [18] (2016)	Case series of 11 patients with acute bulbar palsy as a variant of GBS, 6 of whom had unilateral facial palsy	54 Female	NA	Idiopathic	Dysarthria, diplopia, dizziness, ophthalmoplegia, sensory symptom, gait ataxia, and areflexia	Not mentioned	Not mentioned	Bulbar palsy	IgG anti-GT1a and GQ1b and IgM anti-GT1a and GQ1b	NA	Unremarkable	Abnormal H-reflex, but no details were provided	IVIG	Cured by 7 weeks
Tatsumoto [18] (2016)	Case series of 11 patients with acute bulbar palsy as a variant of GBS, 6 of whom had unilateral facial palsy	26 Female	NA	Diarrhea	Diplopia, dizziness, ophthalmoplegia, sensory abnormality, gait ataxia, and areflexia	Not mentioned	Not mentioned	Bulbar palsy with loss of gag reflux	IgG anti-GT1a and IgM anti-GT1a	NA	Unremarkable	Normal NCS and blink reflexes	IVIG	Cured by 16 weeks
Kim et al. [18] (2016)	Case series of 11 patients with acute bulbar palsy as a variant of GBS, 6 of whom had unilateral facial palsy	21 Male	NA	Idiopathic	Dysarthria, dysphagia, diplopia, dizziness, ophthalmoplegia, and areflexia	Not mentioned	Not mentioned	Bulbar palsy	IgG anti-GT1a and GQ1b	NA	Not done	Normal NCS and blink reflexes	Supportive care only	Cured by 7 weeks
Kim et al. [18] (2016)	Case series of 11 patients with acute bulbar palsy as a variant of GBS, 6 of whom had unilateral facial palsy	20 Male	NA	Diarrhea	Numbness of limbs, diplopia, dizziness, ophthalmoplegia, gait ataxia, and areflexia	Not mentioned	Not mentioned	Bulbar palsy	IgG anti-GQ1b, GT1a, and GM2	NA	Unremarkable	Abnormal H-reflex, but no details were provided	IVIG	Cured by 4 weeks
Kim et al. [18] (2016)	Case series of 11 patients with acute bulbar palsy as a variant of GBS, 6 of whom had unilateral facial palsy	27 Male	NA	Diarrhea	Diplopia, ophthalmoplegia, dizziness, sensory abnormality, gait ataxia, and areflexia	Not mentioned	Not mentioned	Bulbar palsy	IgG anti-GT1a	NA	Albuminocytologic dissociation	Abnormal H-reflex, blink reflexes, and swallowing study, but no details were provided	IVIG	Cured after more than 30 weeks
Tatsumoto^∗^ et al. [19] (2015)	Cross-sectional study evaluated 195 and 68 patients with GBS and MFS, respectively	55 Male	NA	Idiopathic	Tingling of fingertips, right hand weakness, left hand weakness, ataxia, and areflexia	10 days after the symptom's onset	Right	NA	Antiganglioside GM1	Not done	Albuminocytologic dissociation	Compound muscle action potentials had low amplitudes in the median, ulnar, and tibial nerves.	IVIG and IV methylprednisolone	No long-term follow-up was mentioned, but the facial nerve completely recovered after 10 days from the onset
Tan et al.^∗∗^ [20] (2015)	Case series of 4 patients with MFS, one of whom had unilateral facial palsy	57 Female	NA	URTI	Abnormal sensation of the hands and feet, unsteadiness, drooping of the left eyelid, diplopia, slurred speech, dysphagia, and areflexia.	14 days after the symptom's onset	Left	Bulbar palsy	Anti-GQ1b and GT1a	Not done	Albuminocytologic dissociation	Absent median and ulnar sensory nerve action potentials. Motor studies of the median, ulnar, tibial, and peroneal nerves were normal	Plasma exchange.	Extubated on day 16 and underwent rehabilitation. Facial nerve completely recovered, and she was able to ambulate after 44 days of admission. No long-term follow-up was mentioned.
Spagnoli et al. [21] (2015)	Case report and review of the published cases of CMV-related GBS in pediatric population	9 Male	NA	Febrile illness, attributed to CMV infection (+ CMV IgM)	Weakness and paresthesia affecting upper more than lower limbs pain, walking difficulty, and areflexia.	Simultaneous	Right	NA	Anti-GM-2 IgM and low positive anti-GM-1 IgM	Negative	Not done	Acute sensory-motor, mainly axonal polyneuropathy, affecting all limbs, with prevailing sensory findings in the upper limbs and mainly motor in the lower limbs, accompanied by an acute denervation pattern as documented by EMG of the right deltoid muscle.	IV methylprednisolone and IVIG followed by oral deflazacort. Additional oral therapy included alpha-lipoic acid and thiamine which were given on discharge.	On 6-month follow-up, neurological examination was normal
Kamihiro et al. [22] (2011)	Case report	2 Male	NA	Gastroenteritis 6 weeks before admission (rotavirus infection)-URTI 2 weeks before admission	Gait disturbance and areflexia	2 days after the symptom's onset	Left	NA	Negative	Negative	Albuminocytologic dissociation	Diminished CMAP, whereas sensory nerves indicated axonal injury, with unexcitable pattern in one nerve	IVIG	The facial nerve recovered 8 week later.
Lyu and Chen [23] (2004)	Case series of 3 patients with postinfectious acute multiple cranial neuropathy and diagnosed with a variant of GBS, one of whom had unilateral facial palsy	67 Female	NA	URTI	Dizziness, diplopia, ophthalmoplegia, and pain in the orbital region followed by numbness in the hands, nasal speech, and regurgitation of fluids from the nostrils. Normal tendon reflexes.	Simultaneous	Right	Bulbar palsy	Negative	Negative	Albuminocytologic dissociation	Slightly prolonged F-wave latency of the peroneal and tibial nerves with reduced amplitude of sensory nerve action potentials of the median and ulnar nerves.	Supportive care only	The ophthalmoplegia completely recovered by 23 days, but the facial weakness recovery was not mentioned
Sakakibara et al. [24] (2002)	Case report	49 Female	NA	URTI	Neck muscle weakness, dysphagia, weakness and numbness in the upper extremities, respiratory distress, and hyporeflexia on the upper limbs	9 days after symptoms onset	Left	Bulbar palsy and phrenic nerve palsy	Anti-GM1 IgG	Negative	Albuminocytologic dissociation	Median and ulnar nerves showed marked decrease in the compound muscle action potential amplitude after wrist stimulation, but distal latencies and nerve conduction velocities were normal. Absence of F-waves by the stimulation of the median and ulnar nerves, whereas minimal F-wave latency was normal with tibial nerve stimulation.	IVIG.	After a month, respiratory support was taken off. Two months after onset, she was discharged with mild pharyngeal-cervical-brachial weakness and left hypoglossal nerve palsy, but without left diaphragmatic elevation. No long-term follow-up was mentioned
Kamakura et al. [25] (2000)	Case report	23 Female	NA	Diarrhea, sneezing, and headache	Numbness of the extremities, dysphagia, nasal voice, and ataxia. Normal tendon reflexes.	Simultaneous	Right	Bulbar palsy	Anti-GQ1b, GT1a, and low titer of anti GT1b antibody	Negative	Albuminocytologic dissociation	Sensory nerve conduction showed decreased velocity of the sural nerve, and sensory nerve action potential of median and sural nerves was decreased. *F* wave conduction velocities and latencies were normal. A somatosensory evoked potential from the posterior tibial nerve showed delay of latency at N16 and N21.	Plasma exchange	Completely recovered

Abbreviations: Anti-GM, anti-monosialotetrahexosylganglioside; Anti-GT1a, anti-ganglioside GT1a; Anti-GQ1b, anti-ganglioside GQ1b; CMAP, compound muscle action potential; CMV, *Cytomegalovirus*; CN, cranial nerve; CSF, cerebrospinal fluid; EMG, electromyogram; GBS, Guillain–Barré syndrome; IgG, immunoglobulin G; IgM, immunoglobulin M; IV, intravenous; IVIG, intravenous immunoglobulin; MFS, Miller Fisher syndrome; NA, not available; URTI, upper respiratory tract infection; WBCs, white blood cells.

^10 months old.

^∗^The same author reported 5 cases of GBS with delayed unilateral facial palsies, but no details were provided to include them in the table.

^∗∗^The authors diagnosed the patient with MFS overlap with GBS.

## Data Availability

The data that support the findings of this study are available from the corresponding author upon reasonable request.

## Nomenclature

GBS: Guillain–Barré syndrome
BNB: Blood-nerve barrier
CSF: Cerebrospinal fluid
EMG: Electromyography
IVIG: Intravenous immunoglobulin
MRI: Magnetic resonance imaging
MFS: Miller Fischer syndrome
NCS: Nerve conduction study
URTI: Upper respiratory tract infection
WBCs: White blood cells
